# 

**Published:** 1885-08-20

**Authors:** 


### TABLE OF CONTENTS.

ORIGINAL AND SELECTED ARTICLES:

Local Treatment of Carbuncle, by J. S. Geigley, M.D., of Illinois, 281. Strabismus, by W. L. Bullard, of Columbus, Ga., 286. Surgical Dressings, by Edward von Donhoff, M.D., Louisville, Ky., 287. The Treatment of Cholera and Infantum, by W. Byford Ryan, M.D., Indiana, 291. Painless Tooth Extraction, 294 TheLesson Taught by the Epidemic at Plymouth Concerning Typhoid Fever, by M. S. French, M.D., and E. O. Shakespeare, A.M., M.D., Philadelphia, 295. The More Extended Use of Muriate of Cocaine, by A. F. Sampson. M.D., Galveston, Texas, 298. Oleate of Copper in Tinea Capitis, by Robert Boal, M.D., Peoria, Illinois, 302. The Pathology of Cheyne-Stokes Breathing: The Legal Value of the Testimonyof the Insane, 303.

ABSTRACTS AND GLEANINGS:

The Mind-Cure, New and Old, 304. The New Treatment for Typhoid Fever; Malarial Hæmaturia, 305. Induction of Premature Labor, 306. Iodine and Pyrodine, in the Treatment of Asthma; Death Caused by a Purgative in a Case of Internal Occlusion, 307. An Almost Painless Mode of Introducing a Catheter where there is Hyperæsthetic Candition of the Urethra, 308. Overdose of Jaborandi; Non-vesicating Croton Oil; General Statistics of Paralysis, 309. How to Diagnose Gonorrhœa in the Female; The Action of Warm Water Upon the Gravid Uterus; Glycerin as a Tænicide, 310.

SCIENTIFIC ITEMS:

Our National Banking System; The Pain of Being Hung, 311. Hindoo Time, 312.

PRACTICAL NOTES AND FORMULÆ.

Iris and Euonymus; A Specific for Burns; Treatment for Carbuncles; Atropine in Epilepsy, 313.

EDITORIALS AND MISCELLANEOUS.

Editorial Notices; An Aged Doctor; Nocturnal Emissions; A Querry; To Be or Not to Be; That is the Question," 315. Books and Pamphlets Received; Special Notices, 320.

